# High dose sertraline monotherapy fails to protect rhesus macaques from lethal challenge with Ebola virus Makona

**DOI:** 10.1038/s41598-017-06179-y

**Published:** 2017-07-19

**Authors:** Anna N. Honko, Joshua C. Johnson, Jonathan S. Marchand, Louis Huzella, Ricky D. Adams, Nicholas Oberlander, Lisa M. Torzewski, Richard S. Bennett, Lisa E. Hensley, Peter B. Jahrling, Gene G. Olinger

**Affiliations:** 1 Integrated Research Facility, National Institute of Allergy and Infectious Diseases, National Institutes of Health, Frederick, 21702 Maryland USA; 2 BD Technologies/Charles River Labs, Research Triangle Park, 27709 North Carolina USA; 3 MRIGlobal-Global Health Surveillance and Diagnostics, Gaithersburg, Maryland 20878 USA

## Abstract

The recent epidemic of Ebola virus disease in West Africa resulted in an unprecedented number of cases and deaths. Due to the scope of the outbreak combined with the lack of available approved treatment options, there was strong motivation to investigate any potential drug which had existing data reporting anti-Ebola activity. Drugs with demonstrated antiviral activity in the nonhuman primate models already approved for another indication or for which there was existing safety data were considered to be priorities for evaluation by the World Health Organization. Sertraline hydrochloride was reported to have anti-Ebola activity *in vitro* alone and in combination with other approved drugs. Although the efficacy was less than 100% in the murine model, the established safety profile of this product, the potential benefit alone and in combination, as well as the lack of other available options prioritized this compound for testing in the Ebola virus intramuscular rhesus macaque challenge model. Using a blinded dosing strategy, we demonstrated that high dose sertraline monotherapy provided no benefit for the prevention of Ebola virus disease in rhesus macaques with regards to reduction of viral load, morbidity, or survival highlighting the challenges of translating results between *in vitro* and *in vivo* models.

## Introduction

The most recent outbreak of Ebola virus (EBOV) in West Africa resulted in almost 29,000 cases and over 11,000 deaths, more than five times more than all other known Ebola outbreaks combined^1^. While the majority of the outbreak was localized to Guinea, Sierra Leone and Liberia, nations including Germany, Norway, France, Italy, Switzerland, Spain, the United Kingdom and the United States have all treated patients who contracted the virus in West Africa as well as secondary cases, reinforcing that this disease is of international importance.

In 2015 the World Health Organization convened a meeting to review the available data on candidate interventions for Ebola virus in an effort to attempt to prioritize compounds for use in human clinical trials^2^. As a result of that meeting, it was determined that candidate interventions be tiered based on the availability of data. Drugs with known safety profiles and demonstrated efficacy in the nonhuman primate model were determined to be of the highest interest for advancement (Categories A and B), as well as those that had already been administered in *ad hoc* trials or given for compassionate reasons (Category C).

Given the small number of compounds that fell into this category, the next tier were drugs with known safety profiles that were reported to demonstrate anti-Ebola activity *in vitro* or small animal models but for which other data needed to be generated prior to clinical trials (Category D)^2^. Drugs that were already in use and with minimal side effects were considered to be high priority targets for further study. Given these criteria, sertraline was identified as one of the lead drugs in this group.

Selective serotonin reuptake inhibitors (SSRIs) are a class of compounds typically prescribed for use as antidepressants for mood or anxiety disorders. Additionally, SSRIs have previously been reported to have antiviral effects via either direct or off-target mechanisms. Several studies and meta-analyses have linked pro-inflammatory responses in development of mood disorders and suggested the effects of SSRIs could be attributed to anti-inflammatory effects of the drugs^3–9^. Sertraline has been shown to reduce influenza-induced lung inflammation and mortality when combined with phosphodisestrase-4 inhibitor in a mouse model, providing evidence that SSRIs may have benefit in treatment of viral disease^10^ in synergy with other drugs. The SSRI citalopram was shown to enhance *ex vivo* natural killer and CD8 cell responses as well as reduce HIV infectivity of macrophages^11^. Anti-HIV effects were further supported in a clinical study in which HIV patients treated with citalopram, sertraline and trazodone SSRIs all presented with reduced viral loads in the central nervous system^12^.

Sertraline has been reported to have antiviral efficacy against filoviruses *in vitro* ^13, 14^. Additionally, in the mouse-adapted EBOV model, partial protection of C57BL/6 mice was observed when mice were treated with 10 mg/kg sertraline twice daily for 10 days beginning 1 hour post-exposure^13^. The authors postulated the antiviral effect may result from inhibition of virus trafficking to NPC1+ endosomes by indirectly inhibiting the function of acid sphingomyelinase^13^, thought to be required for virus entry^15^, or effects on the physiochemical properties of the endosome membrane^14^. NPC1 has previously been shown to be indispensable cellular receptor for virus entry following glycoprotein processing by endosomal cysteine proteases^16, 17^. Both structure-property-activity relation modeling^18^ and mouse models of cystic fibrosis^19^ have demonstrated predicted and functional inhibitory effects of sertraline on ASM function. Collectively, these data support a possible mechanism for direct anti-EBOV effects of sertraline.

The apparent efficacy of sertraline as an antiviral therapeutic for Ebola virus *in vitro*, coupled with a long half-life^20, 21^ and good tolerability profile^22^ warranted further evaluation of the drug in the nonhuman primate model of Ebola virus. This study evaluated whether high dose sertraline treatment alone would provide survival benefit, a reduction in viral loads or amelioration of clinical disease in rhesus macaques challenged with a lethal dose of Ebola virus.

## Results

### High dose treatment with sertraline does not protect rhesus macaques from subsequent challenge with a lethal dose of Ebola virus Makona

**Figure 1:**
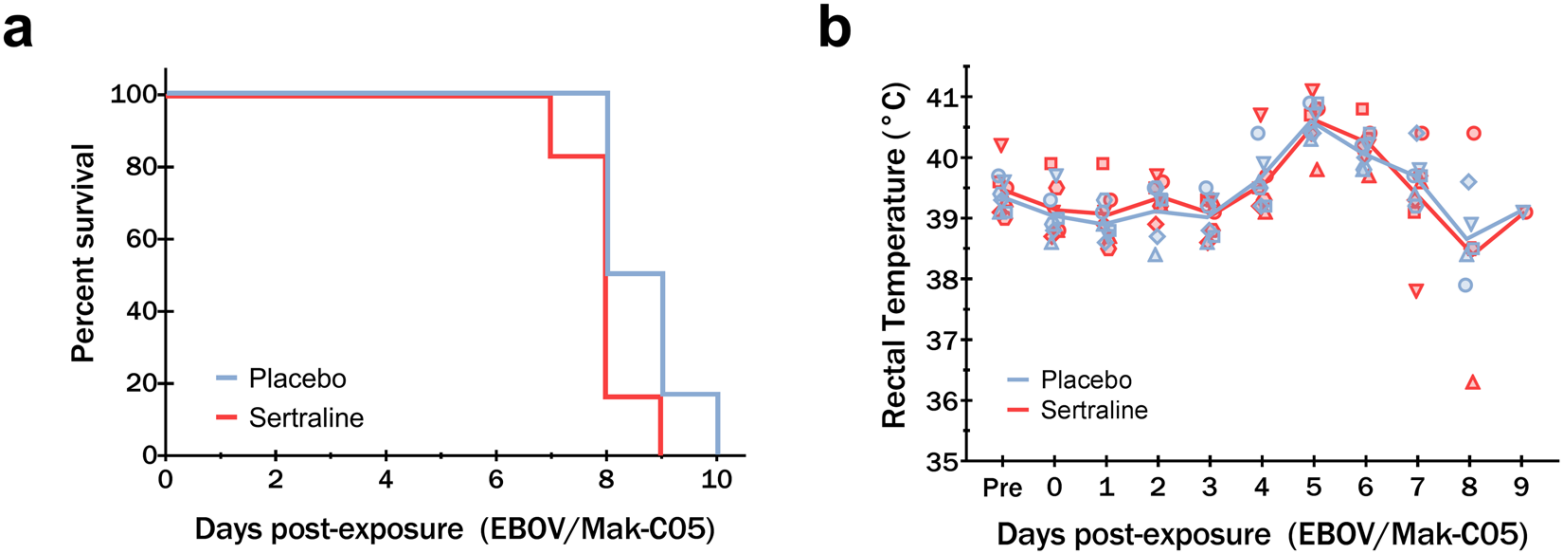
Survival analysis of sertraline and placebo treated animals exposed to a lethal dose of Ebola virus. Groups of 6 male rhesus macaques were treated with 200 mg sertraline hydrochloride (red) or placebo (blue) by orogastric tube daily beginning six days prior to intramuscular challenge with EBOV/Mak-C05. The survival analysis (**a**) shows a median time-to-disposition of 8 and 8.5 days for sertraline- and placebo-treated groups, respectively. Rectal temperatures (**b**) for sertraline hydrochloride and placebo treated animals were evaluated at each anesthetized physical.

Beginning six days prior to challenge and daily throughout, two groups of six male rhesus macaques received either 200 mg of sertraline hydrochloride reformulated into Prima Treat tablets, or tablets without drug as placebo controls. Treatments were administered once daily in the morning via orogastric tube while animals were sedated. Animals were challenged intramuscularly with a uniformly lethal dose of EBOV/Mak-C05 (1090 PFU) and monitored daily for development of clinical signs of disease. Despite daily treatment, all animals treated with sertraline hydrochloride or placebo were euthanized or succumbed to disease with median times to disposition of 8 and 8.5 days, respectively. The survival curve is depicted in Fig. 1. There was no statistically significant difference median time-to-disposition between the treated and placebo groups as measured by the Log-rank test (p = 0.1405).

### Treatment does not diminish clinical or pathological disease associated with Ebola virus hemorrhagic fever

All macaques developed progressive disease consistent with a lethal dose of Ebola virus, irrespective of treatment. Animals became uniformly febrile (rectal temperature >39.7 °C) between days 4 and 5 post-exposure as shown in Fig. 1, followed by rapid decline just prior to death. Normal and stimulated activity as well as responsiveness were used as the primary criteria for assessing early endpoint criteria. Primary responsiveness scores for nonhuman primates are depicted in Table 1. A reduction in overall activity and responsiveness was seen by day 6 in both treated and placebo groups. This was accompanied by other early clinical indicators of disease, including reduced appetite, and lymphadenopathy in the lymph nodes proximal to the virus inoculation site. Clinical status of the nonhuman primates in both sertraline treated and placebo groups rapidly deteriorated from day 7 until terminal disposition, with a dramatic reduction in activity and responsiveness with associated recumbence and/or prostration, weakness, moderate to severe dehydration and anorexia as well as the development of characteristic petechial or maculopapular rash.

**Table 1:** Clinical responsiveness scores for study subjects.

Day PE	Placebo						Sertraline					
	NHP 1	NHP 2	NHP 3	NHP 4	NHP 5	NHP 6	NHP 1	NHP 2	NHP 3	NHP 4	NHP 5	NHP 6
−7	0	0	0	0	0	0	0	0	0	0	0	0
0	0	0	0	0	0	0	0	0	0	0	0	0
1	0	0	0	0	0	0	0	0	0	0	0	0
2	0	0	0	0	0	0	0	0	0	0	0	0
3	0	0	0	0	0	0	0	0	0	0	0	0
4	0	0	0	0	0	0	0	0	0	0	0	0
5	0	0	0	0	0	0	0	0	0	0	0	0
6	0	1	1	1	2	1	1	1	1	1	2	2
7	1	1	2	2	2	1	1	3	2	3	2	3
8	**3**	2	2	**3**	**4**	2	2	**S**	**4**	**S**	**3**	
9		**S**	**4**			2	**4**					
10						**S**						

Cage-side unanesthetized responsiveness was scored on the following 5-point range, where a score of 4 met primary endpoint criteria and 3 initiated secondary criteria evaluation: 0 = alert, responsive, normal activity, free of disease signs or exhibits only resolved/resolving disease signs; 1 = slightly diminished general activity, subdued but responds normally to external stimuli; 2 = withdrawn, may have head down, or fetal posture, or hunched, or reduced response to external stimuli; 3 = recumbent but able to rise if stimulated, or moderate to dramatically reduced response to external stimuli; 4 = persistently recumbent, or severely or completely unresponsive, or may have signs of respiratory distress. Bold numbers indicate terminal time points where animals were euthanized. “S” indicates animals succumbed prior to euthanasia.

Necropsies were performed on all animals, with pathological findings that were generally consistent across all animals regardless of treatment. Rash was observed on the haired skin of the face, ears, axillary and inguinal region and evidence of multi-organ system damage noted including enlargement, discoloration of hepatic and renal tissue, and enlargement and friability of splenic tissue. Additionally, the adrenal glands were congested or hemorrhagic in the majority of the animals. Hemorrhage, congestion and edema were noted in both the gastrointestinal tract and peripheral lymph nodes. Clearly demarcated congestion of the gastroduodenal junction was observed in half of the animals, a distinctive feature of EBOV in macaques. Severity and presentation of pathological findings were equivalent in both treated and untreated animals.

### Animals treated with high dose sertraline present with aberrant hematological and biochemical abnormalities consistent with Ebola virus disease

**Figure 2:**
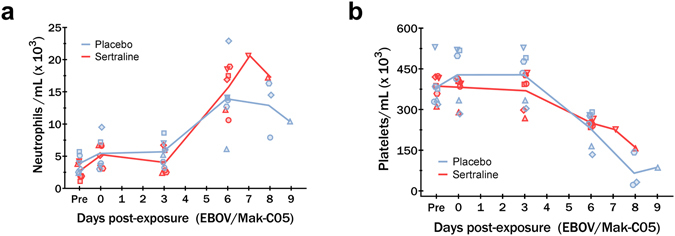
Neutrophil and platelet counts for sertraline hydrochloride and placebo treated animals. Pronounced neutrophilia and thrombocytopenia was observed in all animals by day 6 post exposure. Animals were treated with 200 mg sertraline hydrochloride (red) or placebo (blue) by orogastric tube. Group means are presented as lines, with individual values depicted as shapes.

Complete blood counts with differential and general chemistry panels were performed at scheduled sampling points and euthanasia following exposure of nonhuman primates to Ebola virus. Hematology profiles revealed a concomitant neutrophilia and substantial reduction in platelets at day 6 and terminal time points post-exposure (Fig. 2). A mild drop in red blood cells with associated decline in hemoglobin and hematocrit consistent with hemorrhagic disease was also observed beginning day 3 post exposure (data not shown). These findings were observed equivalently in both sertraline and placebo treated animals.

**Figure 3:**
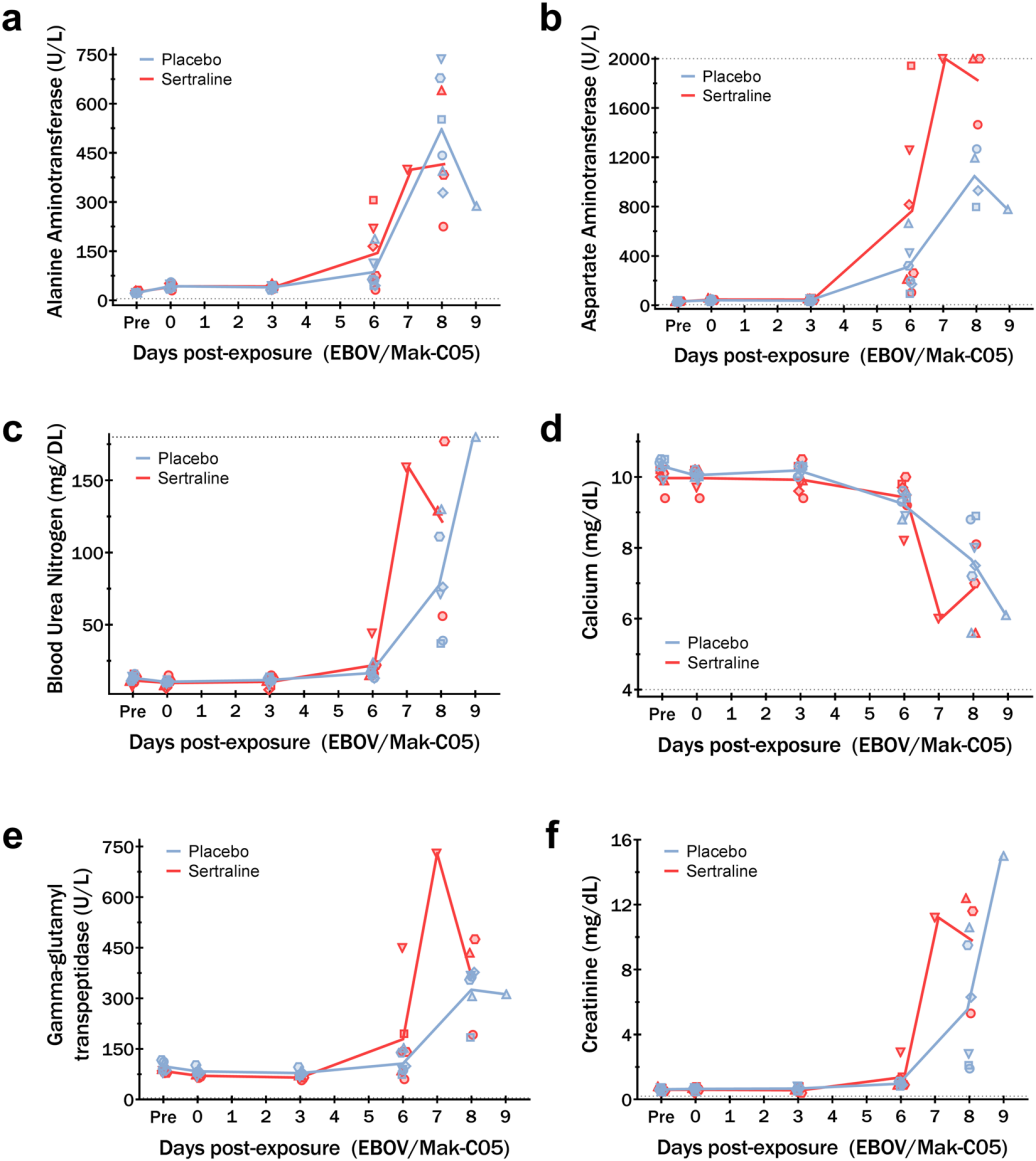
Serum chemistry profiles for sertraline hydrochloride- and placebo-treated macaques. All animals presented with substantial elevation in serum transaminases (**a**,**b**), alkaline phosphatase (not shown) and gamma-glutamyl transpeptidase (**e**) by day 6 post-exposure. Enzyme levels remained elevated until death. All animals exhibited renal dysfunction with elevated blood urea nitrogen (**c**) and creatinine (**f**) with concomittant decrease in serum total calcium (**d**). Animals were treated with 200 mg sertraline hydrochloride (red) or placebo (blue) by orogastric tube. Group means are presented as lines, with individual values depicted as shapes. Upper or lower dynamic ranges of analytes are shown by dotted lines as applicable.

As expected, exposure to Ebola virus also resulted in extensive biochemical abnormalities. Significant elevation of serum transaminases, gamma glutamyl-transpeptidase, (depicted in Fig. 3) as well as alkaline phosphatase and total bilirubin (not shown) were observed as early as day 6 post-exposure. Both drug and placebo treated animals also presented with significantly impaired renal function as measured by markedly elevated blood urea nitrogen and creatinine levels (Fig. 3), hypoalbuimaemia (not shown) and greatly reduced total serum calcium (Fig. 3).

### High dose Sertraline treatment does not reduce virus load in Ebola virus challenged rhesus macaques

**Figure 4:**
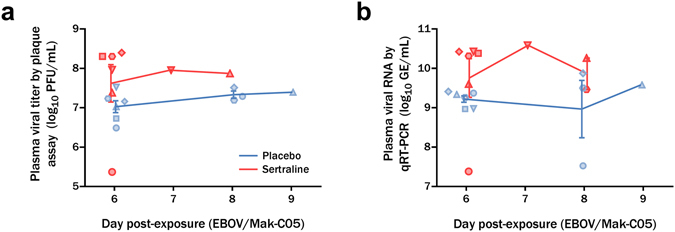
Circulating viremia of sertraline and placebo treated animals exposed to a lethal dose of Ebola virus. Animals were treated with 200 mg sertraline hydrochloride (red) or placebo (blue) by orogastric tube. Log_10_ plasma viremia levels as measured by plaque assay (**a**) and qRT-PCR (**b**) are shown as group means with SEM. Individual values are depicted as shapes. By day 6 post-exposure, animals exhibited substantial viremia that persisted until death. Statistical analysis showed no significant difference between placebo and sertraline treatment at D6.

Virus load was quantified using both quantitative real-time PCR (qRT-PCR) targeting the virus and titration by plaque assay. Results are shown in Fig. 4. Circulating virus was detectable by day 6 post-exposure, correlating with onset of overt clinical signs. Infectious virus titers exceeded 10^7^ plaque-forming units (PFU)/mL for treated animals, except for a single sertraline treated animal for which a terminal sample was not available. qRT-PCR results mirrored plaque assay findings. The magnitude and kinetics of viremia were similar in sertraline and placebo treated animals and treatment with sertraline hydrochloride provided no reduction in either infectious virus or viral genome copies.

Statistical analysis was performed to compare the viral load data between the placebo and sertraline groups on Day 6. Lack of matching data points from later timepoints prevented formal statistical comparisons between groups on days 7–9. The t-test for the plaque assay data (Fig. 4a) compared the mean log10 viral load in placebo group on Day 6 of 9.22 and the mean log_10_ viral load in treatment arm of 9.75 for a mean difference of −0.534. The 85% confidence interval is (−1.796, 0.726). The P value for the two arms comparison was 0.3297. Similarly, the t-test for the qRT-PCR data (Fig. 4b) compared the mean log_10_ viral load in the placebo group on Day 6 of 7.025 to the mean log_10_ viral load in the sertraline treatment arm of 7.619 for a mean difference of −0.593. The 85% confidence interval was (−1.813, 0.626). The P value for the two arms comparison is 0.2789. From these analyses, neither result was significant.

## Discussion

Therapeutic drug repurposing or repositioning is one method that sponsors utilize to more rapidly integrate potential treatments into clinical care. Existing drugs with known safety profiles are repositioned for new indications, potentially bypassing the bottlenecks associated with traditional pharmaceutical development when interventions can be implemented without significantly altering approved dosing regimens. This reduces risk, as lack of an established safety profile in preclinical and clinical phases is a reasons for failure of novel compounds, and also potentially shortens the path to review by the Food and Drug Administration by building on the foundation of existing research. Although there is value in this strategy, particularly in an outbreak situation when time is critical and resources limited, the challenge is to appropriately evaluate and prioritize therapeutic options.

These results were disappointing in that we expected at least a partially beneficial effect from use of sertraline in the EBOV macaque model based on promising *in vitro* and rodent efficacy data. Reasonable expectations included prolonged time to death, reduced viremia and clinical signs, and possibly increased survival and had such results been obtained, sertraline use might have been considered in the outbreak situation. Nevertheless, these negative results highlight the concern about extrapolating from *in vitro* and rodent data to nonhuman primates and humans, especially for other cationic amphiphile compounds postulated to have similar mechanisms of action such as amiodarone, clomiphene and toremifene. We also feel that it is important to publish negative data lest other investigators be tempted on the basis of preliminary data to unknowingly repeat the nonhuman primate studies^23^. The implementation of randomization, use of a blinding strategy for prevention of bias, and statistically-supported sample sizes allowed for confidence in the conclusion, indisputably vital for translational science and feasible even in biocontainment facilities^24, 25^.

These data might be utilized to learn more about the mechanisms of action that are dissimilar between the *in vitro* and *in vivo* models to optimize the regimen^23^. Although the objective of this study was to evaluate the efficacy of high dose sertraline hydrochloride as a prophylactic preventative of Ebola virus in nonhuman primates, it remains possible that sertraline used synergistically with other drugs that have demonstrated efficacy may still prove valuable for combination treatment of EBOV disease. There are a number of variables that impact the translational failure from early *in vitro* and preclinical work to final clinical benefit^26–28^, and although the safety profile is generally a good predictor regarding translation of toxicity, other important considerations may include reproducibility, accuracy of recapitulating aspects of disease, or similarities of the host inflammatory reactions or immune response.

Route and bioavailablilty are important considerations when performing *in vivo* drug studies. Factors such as the type of formulation (whether research-grade or pharmaceutical), drug-drug interactions or even interspecies differences in gastrointestinal adsorption may have effects^29^. In this study, we evaluated pharmaceutical drug that was compounded into tablets and prepared for oral dosing, which theoretically is more aligned with how prophylactic patient dosing would occur. In contrast, the prior mouse experiment utilized a potentially less physiological route of dosing (intraperitoneal) following the same route of challenge, using stock sertraline prepared in a DMSO/phosphate-buffered saline solution^13^.

In the 25 years since the FDA licensure of sertraline, it has been demonstrated to be both broadly effective for treatment of acute and long-term mood disorders and found safe and well-tolerated in various patient populations^22^ with very low likelihood for overdosage^21^. Sertraline is slowly absorbed following oral dosing, and steady-state is reached after approximately a week. Typical adult clinical doses of sertraline would include an initial period of 50 mg per day followed by maintenance doses ranging from 50 to 200 mg per day^20–22^. Available cynomolgus data of oral sertraline dosing up to 20 mg/kg reported circulating plasma concentrations within clinical ranges when sampled at a week^30^. Our original dosing rationale was 50 mg per NHP as estimated from simple allometric scaling calculations. Taking the above factors into consideration, we increased our dose four-fold to 200 mg/day (median of 55 mg/kg, range 51–57 mg/kg). The intent was to both mimic typical clinical label indications, as well as use a prophylactic dosing strategy as hypothesized might be sufficient with a lower circulating *C*_max_ (maximum concentration) with potential accumulation in target tissues^13^. Subsequent to our efficacy study, a single-dose oral PK study performed in rhesus macaques (data not shown) at 2 mg/kg and 50 mg/kg demonstrated that sertraline was well-tolerated and both sertraline and the metabolite desmethylsertraline were slowly eliminated from circulation. Although the plasma *C*_max_ for the for the 50 mg/kg single-dose PK of sertraline (mean 179 ng/mL, range 129–267 ng/mL) did not reach estimated *in vitro* IC_50_ levels of approximately 2 *μ*M^13^, it is expected that after multiple days of dosing accumulation of sertraline/desmethylsertraline along with the extended plasma elimination phase of the compounds would provide sufficient levels of bioavailability.

Recently, there has been debate regarding the stringency of the rhesus macaque model for evaluation of filoviruses, with concerns that as the most stringent model it is possible that some countermeasures may be missed. When assessing the appropriateness of animal models for selection, a number of aspects must be considered to align the characteristics of the disease that fit most closely as determined by the specific research question^31^. The balance of the advantages and disadvantages posed by each model, the aspects of disease recapitulated by the system or model, and an understanding of the limitations of the models are all critical. Rodent models of EBOV are attractive for evaluating the efficacy of antiviral treatments, however, success in mouse and guinea pig models has not always reproduced in nonhuman primates^25, 32, 33^. The rodent models require either adapted virus, or use of humanized or immune-deficient animals, and these models also fail to recapitulate the full spectrum of human disease.

Although considered stringent, the rhesus macaque model for EBOV disease is frequently reported as one of the highest standards for evaluation of therapeutics^25, 32^ due to the similarities of the physiology, immune system responses and homology shared between the macaque and humans. At the time this experiment was performed the rhesus macaque model was being used as a proposed recommendation criteria for the advancement of candidate products to clinical trials. It remains unclear how IC_50_ values from *in vitro* assays correlates to protective levels in animal models. Compounds that have recently demonstrated promising efficacy in nonhuman primate models of EBOV with known correlative pharmacokinetics demonstrated drug exposures in nonhuman primates greatly exceeding the submicromolar IC_50_^34, 35^, but additional studies will be needed to understand these relationships.

In summary, despite promising evidence both *in vitro* and in the murine model of EBOV, prophylactic treatment with sertraline hydrochloride did not provide survival benefit in rhesus macaques challenged with a lethal dose of EBOV. The pharmacokinetics for sertraline observed in the rhesus PK study suggest that drug exposure modeled for the efficacy study was similar in range to typical clinical doses used in humans^20^. Collectively, these results suggest high dose sertraline alone does not provide benefit for preventing or reducing clinical severity of in the nonhuman primate model and monotherapy is likely of little therapeutic benefit for humans infected with EBOV. Although the objective of this study was to evaluate the efficacy of high dose sertraline hydrochloride as a prophylactic preventative for Ebola virus in nonhuman primates, it remains possible that sertraline used in combination with other drugs that have demonstrated efficacy may still prove valuable for combination treatment of EBOV disease. In addition, the data generated in these studies will be useful for future efforts to begin to define how model systems predict performance between and across systems.

## Materials and Methods

### Ethics statement and disclosures

Research was performed in accordance with animal study protocols approved by the Animal Care and Use Committee (ACUC) of the National Institute of Allergy and Infectious Diseases (NIAID) Division of Clinical Research (DCR), part of the National Institutes of Health (NIH). Protocols adhere to the recommendations stated in *The Guide for the Care and Use of Laboratory Animals* and were developed in compliance with the United States Department of Agriculture (USDA) Animal Welfare Act regulations, U.S. Public Health Service Policy on Humane Care and Use of Laboratory Animals, NIH policies and guidelines, and other federal statutes and regulations relating to animals and experiments involving animals. The NIAID Integrated Research Facility (IRF)-Frederick, where the research was conducted, is fully accredited by the Association for Assessment and Accreditation of Laboratory Animal Care, International (AAALACi).

### Biosafety

All work with infectious EBOV and potentially infectious materials derived from animals was conducted in a Biosafety Level 4 (BSL 4) laboratory in the NIAID IRF-Frederick.

### Virus source and propagation

The C05 isolate of Ebola Makona (full designation: Ebola virus/H.sapiens-tc/GIN/2014/Makona-C05, abbreviated name: EBOV/Mak-C05, GenBank accession number KP096420.1, BioSample number SAMN03611815)^36^, was generously provided by Dr. Kobinger of Public Health Agency Canada. This Vero E6 p1 stock was further propagated for two additional passages in VeroE6 cells (obtained through BEI Resources, NIAID, NIH: VERO C1008 (E6), Kidney (African green monkey), Working Cell Bank, NR-596) in Minimum Essential Medium (MEM)-*α*, GlutaMAX,TM no nucleosides (Gibco, ThermoFisher Scientific) supplemented with 2% US-origin, certified, heat-inactivated fetal bovine serum (HI-FBS, Gibco, ThermoFisher Scientific) to yield a master working bank and a subsequent working virus stock. Following harvest, HI-FBS was QS’d to 10% final concentration before cryopreservation. The virus working stock used for exposure (GenBank accession no. KX000400.1, BioSample: SAMN04490241, internal reference IRF0137) was predominantly of the 7U genotype (98.9963%) as determined by Next Gen Sequencing.

### Formulation and preparation of sertraline hydrochloride

Sertraline hydrochloride (Zoloft, 50 mg tablets) was obtained from the NIH pharmacy and reformulated by Bio-Serv (Flemington, NJ, USA) in the form of PRIMA-Treats containing 100 mg of sertraline hydrochloride per tablet. PRIMA-Treats without drug were used as placebo control. Tablets were pulverized to a fine powder and immediately prior to treatment suspended in 30 mL PediaSure (Abbott) for orally treating each animal. Animals were provided 10 mg/kg metoclopramide ten to fifteen minutes prior to dosing as an antiemetic agent to prevent vomiting.

### Treatment, challenge and observation of study animals

Twelve male rhesus macaques of Indian origin, aged 2–3 years, were randomized into two balanced groups based on age and weight. Group identities remained blinded to study personnel conducting work until study completion. Animals were pre-treated daily with either 200 mg of sertraline hydrochloride (median of 55 mg/kg, range 51–57 mg/kg) or placebo starting six days prior to virus exposure through the end of study. Treatment was provided orally through orogastric tube to the stomach under anesthesia. On study day 0, animals were anesthetized and challenged intramuscularly (IM) with a target dose of 1000 plaque-forming units (PFU) in the triceps muscle (actual dose = 1090 PFU). Animals were observed at least once daily until development of overt clinical signs of disease, after which frequency of observations were increased. To obtain objective data, early endpoints were evaluated using a statistically-established euthanasia criteria as previously reported^37^ based on combined observed responsiveness criteria and secondary chemistry criteria.

### Hematology and serum chemistry

Complete blood count with leukocyte differential was performed from peripherally collected blood samples using Vacuette K3EDTA tubes and a Sysmex XT-2000iV hematology instrument using a preprogrammed monkey species profile (Sysmex America, NY). Plasma and serum were prepared by separation for 10 minutes at ambient temperature with centrifuge set to 1800 RCF. Serum chemistries were performed using the Piccolo Xpress Analyzer and General Chemistry 13 discs (Abaxis, Abbott Point of Care, NJ) from Vacuette Z Serum Clot Activator tubes (Greiner Bio-One, Monroe, NC).

### Viral load evaluation

#### Viral load determination using RT-PCR

Quantitative real time RT-PCR Separated K3EDTA plasma was inactivated in TRIzol LS (ThermoFisher Scientific, Waltham, MA) in accordance with manufacturer’s instructions. RNA was isolated using an extraction method developed by the United States Army Medical Research Institute of Infectious Diseases^38^. Briefly, 70 *μ*L of TRIzol LS-inactivated sample was added to 280 *μ*L of QIAGEN Buffer AVL containing carrier RNA and extracted using the QIAamp Viral RNA Mini Kit in accordance with manufacturer’s instructions (QIAGEN, Germantown, MD). Sample was eluted in 70 *μ*L of Buffer AVE, aliquoted and frozen until assay. Plasma virus RNA load was measured using BEI Resources Critical Reagents Program EZ1 RT-PCR (TaqMan) assay kit on an ABI 7500 FastDx (Applied Biosystems, ThermoFisher Scientific)^39^ in accordance with manufacturer’s instructions using a synthetic RNA standard curve and reported as gene copies (GC) per mL of sample.

#### Virus titration by plaque assay

Infectious dose and plasma virus load were measured by a modified plaque assay utilizing an Avicel RC 591 semi-solid overlay. Briefly, VeroE6 cells were plated in 6-well plates for target confluence of 90–100% on the day of assay and maintained in Gibco Minimum Essential Medium (MEM)-*α* with GlutaMAXTM and 5% HI-FBS. Media was aspirated from wells and 10-fold dilutions of samples were titrated in at least triplicate in 300 *μ*L inoculum volume. Samples were adsorbed to the cell monolayers for 1 hour ±10 minutes at 37 °C, 5% CO_2_ with gentle rocking approximately every 15 minutes to prevent monolayer drying. A 1:1 overlay of 2.5% Avicel RC 591 biopolymer (FMC BioPolymer) mixed with 2X Modified Eagle Medium (Temin’s modification, Gibco) supplemented with 2X Antibiotic-Antimycotic (Gibco), 2X GlutaMAX (GibcoTM) and 10% HI-FBS (Gibco). Cells were incubated at 37 °C, 5% CO_2_ for 8 days, stained and fixed with 0.2% aqueous Gentian Violet (Ricca Chemicals) prepared in 10% neutral buffered formalin (Richard-Allan Scientific) for 30 minutes, rinsed and enumerated.

### Data availability

The virus working stock used for exposure (GenBank accession no. KX000400.1, BioSample: SAMN04490241).

## References

[1] World Health Organization. Ebola Situation Report March 30, 2016. http://apps.who.int/ebola/current-situation/ebola-situation-report-30-march-2016, doi:arXiv:1011.1669v3 (2016).

[2] World Health Organization. Categorization and prioritization of drugs for consideration for testing or use in patients infected with Ebola. www.who.int/medicines/ebola-treatment/2015_0703TablesofEbolaDrugs.pdf? ua=1{&}ua=1 (2015).

[3] Black C, Miller BJ (2015). Meta-analysis of cytokines and chemokines in suicidality: Distinguishing suicidal versus nonsuicidal patients. Biol. Psychiatry.

[4] Valkanova V, Ebmeier KP, Allan CL (2013). CRP, IL-6 and depression: A systematic review and meta-analysis of longitudinal studies. J. Affect. Disord..

[5] Jiang H-Y (2014). Specific serotonin reuptake inhibitors prevent interferon-alpha-induced depression in patients with hepatitis C: a meta-analysis. Clin. Gastroenterol. Hepatol..

[6] Liu Y, Ho RC-M, Mak A (2012). Interleukin (IL)-6, tumour necrosis factor alpha (TNF-alpha) and soluble Interleukin-2 receptors (sIL-2R) are elevated in patients with major depressive disorder: A meta-analysis and meta-regression. J. Affect. Disord..

[7] Hannestad J, DellaGioia N, Bloch M (2011). The effect of antidepressant medication treatment on serum levels of inflammatory cytokines: a meta-analysis. Neuropsychopharmacology.

[8] Sarkar S, Schaefer M (2014). Antidepressant pretreatment for the prevention of interferon alfa-associated depression: A systematic review and meta-analysis. Psychosomatics.

[9] Ehret M, Sobieraj DM (2014). Prevention of interferon-alpha-associated depression with antidepressant medications in patients with Hepatitis C virus: A systematic review and meta-analysis. Int. J. Clin. Pract..

[10] Sharma G (2013). Reduction of influenza virus-induced lung inflammation and mortality in animals treated with a phosophodisestrase-4 inhibitor and a selective serotonin reuptake inhibitor. Emerg. Microbes Infect..

[11] Benton T (2010). Selective serotonin reuptake inhibitor suppression of HIV infectivity and replication. Psychosom. Med..

[12] Letendre SL (2007). The role of cohort studies in drug development: clinical evidence of antiviral activity of serotonin reuptake inhibitors and HMG-CoA reductase inhibitors in the central nervous system. J. Neuroimmune Pharmacol..

[13] Johansen LM (2015). A screen of approved drugs and molecular probes identifies therapeutics with anti-Ebola virus activity. Sci. Transl. Med..

[14] Kouznetsova J (2014). Identification of 53 compounds that block Ebola virus-like particle entry via a repurposing screen of approved drugs. Emerg. Microbes Infect..

[15] Miller ME, Adhikary S, Kolokoltsov AA, Davey RA (2012). Ebolavirus requires acid sphingomyelinase activity and plasma membrane sphingomyelin for infection. J. Virol..

[16] Miller EH (2012). Ebola virus entry requires the host-programmed recognition of an intracellular receptor. EMBO J.

[17] Herbert AS (2015). Niemann-pick C1 is essential for ebolavirus replication and pathogenesis *in vivo*. MBio.

[18] Kornhuber J (2008). Identification of new functional inhibitors of acid sphingomyelinase using a structure-property-activity relation model. J. Med. Chem..

[19] Becker KA (2010). Acid sphingomyelinase inhibitors normalize pulmonary ceramide and inflammation in cystic fibrosis. Am. J. Respir. Cell Mol. Biol..

[20] Mandrioli R, Mercolini L, Raggi MA (2013). Evaluation of the pharmacokinetics, safety and clinical efficacy of sertraline used to treat social anxiety. Expert Opin. Drug Metab. Toxicol..

[21] DeVane, C. L., Liston, H. L. & Markowitz, J. S. Clinical pharmacokinetics of sertraline (2002). 10.2165/00003088-200241150-00002 12452737

[22] Sheehan DV, Kamijima K (2009). An evidence-based review of the clinical use of sertraline in mood and anxiety disorders. Int. Clin. Psychopharmacol..

[23] London AJ, Kimmelman J (2015). Why clinical translation cannot succeed without failure. Elife.

[24] van der Worp HB (2010). Can animal models of disease reliably inform human studies?. PLoS Med..

[25] Geisbert TW, Strong JE, Feldmann H (2015). Considerations in the use of nonhuman primate models of Ebola virus and Marburg virus infection. J. Infect. Dis..

[26] Ioannidis JPA (2005). Why most published research findings are false. PLoS Med..

[27] Chalmers I, Glasziou P (2009). Avoidable waste in the production and reporting of research evidence. Lancet.

[28] McGonigle P, Ruggeri B (2014). Animal models of human disease: Challenges in enabling translation. Biochem. Pharmacol..

[29] Pelkonen O, Boobis AR, Gundert-Remy U (2001). *In vitro* prediction of gastrointestinal absorption and bioavailability: an experts’ meeting report. Eur. J. Clin. Pharmacol..

[30] Shively CA, Register TC, Higley JD, Willard SL (2014). Sertraline effects on cerebrospinal fluid monoamines and species-typical socioemotional behavior of female cynomolgus monkeys. Psychopharmacology (Berl)..

[31] Louz D, Bergmans HE, Loos BP, Hoeben RC (2013). Animal models in virus research: their utility and limitations. Crit. Rev. Microbiol..

[32] Bente D, Gren J, Strong JE, Feldmann H (2009). Disease modeling for Ebola and Marburg viruses. Dis. Model. Mech..

[33] Geisbert, T. W. *et al*. Evaluation in nonhuman primates of vaccines against Ebola virus (2002). 10.3201/eid0805.010284 PMC3369765 11996686

[34] Warren TK (2014). Protection against filovirus diseases by a novel broad-spectrum nucleoside analogue BCX4430. Nature.

[35] Warren TK (2016). Therapeutic efficacy of the small molecule GS-5734 against Ebola virus in rhesus monkeys. Nature.

[36] Hoenen T (2014). Complete genome sequences of three Ebola virus isolates from the 2014 outbreak in West Africa. Genome Announc..

[37] Warren TK (2014). Euthanasia assessment in ebola virus infected nonhuman primates. Viruses.

[38] Shan, S., Geisler, K., Honko, A. & Ingram, M. RNA Extraction of Ebola Zaire in TRI Reagent LS Using Various Protocols (2012).

[39] Naval Medical Research Center. Ebola Zaire (EZ1) rRT-PCR (TaqMan(R)) Assay On ABI(R) 7500 Fast Dx, LightCycler(R) and JBAIDS (2014).

